# Secondary hypertension due to isolated interrupted aortic arch in a 45-year-old person: A case report

**DOI:** 10.1097/MD.0000000000009122

**Published:** 2017-12-08

**Authors:** Jian Mei Zhou, Xin Wen Liu, Yi Yang, Bo Zhong Wang, Jian An Wang

**Affiliations:** a Hypertension Center of Zhejiang Hospital; b Heart Center of the Second, Affiliated Hospital of Zhejiang University School of Medical, Hangzhou, Zhejiang Province, China.

**Keywords:** 45-year-old person, isolated interrupted aortic arch, secondary hypertension

## Abstract

**Rationale:**

Though it is rare, isolated interrupted aortic arch (IAA) could lead to hypertension. Surgical repair is the only effective curative method to treat IAA conditions and patients with IAA can hardly survive to adulthood with medication alone. We report an IAA case that of a 45-year-old male patient who survived for 45 years without surgical treatment.

**Patient concerns:**

A 45-year-old man was referred to the hospital presenting with abnormal blood pressure level. Both computed tomography angiogram (CTA) and angiography revealed IAA.

**Diagnoses:**

The patient was diagnosed as IAA based on computed tomography angiogram (CTA) and angiography.

**Interventions:**

The patient's blood pressure was severely high and refractory. He refused surgical treatment and accepted antihypertensive medication for 10 days.

**Outcomes:**

The patient's office blood pressure level was abnormal, fluctuating between 140/90 and 160/100 mm Hg, but 24-hour ambulatory blood pressure monitoring showed normal level.

**Lessons:**

Hypertension due to IAA could be controlled with medications, even surgery is not performed. The discrepancy between ambulatory and office blood pressure levels may be due to the white coat effect.

## 1 Introduction

IAA is a very rare congenital malformation defined as a complete loss of anatomical continuity between ascending and descending portions of the aorta. It occurs in 3 per million live births and accounts for <1% of all congenital heart disorders.^[1]^ IAA is a rare secondary cause of hypertension. It is usually detected in the prenatal period or during infancy with high mortality, if without surgical repair,^[2–5]^ which is rare in adulthood.

## 2 Methods

We report the case study. The committee waived the requirement for approval for a single case study with medical records. Informed consent was given.

## 3 Case presentation

**Figure 1:**
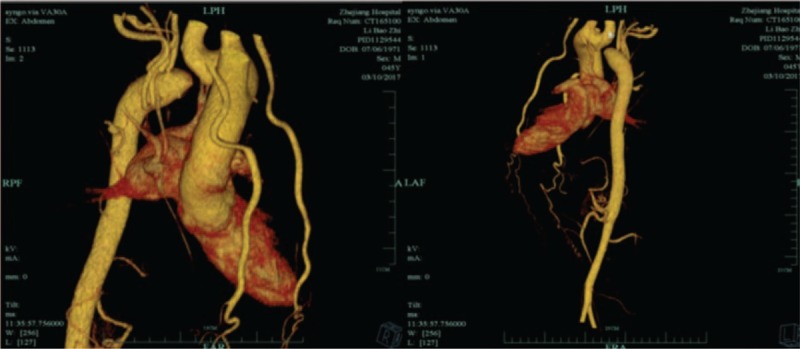
Computed tomography angiogram (CTA) revealed a type A interrupted aortic arch with complete discontinuity of the aortic lumen distal to the origin of the left subclavian artery, and a massive aorta arch which included 2 full-fledged collaterals network ensuring blood circulation to distal aorta.

**Figure 2:**
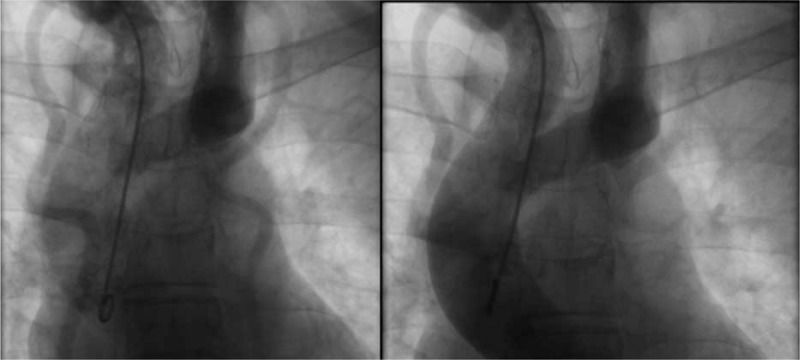
Angiography showed absence of anatomical continuity between the ascending aorta distal to the left subclavian artery and the descending aorta. There was no patent ductus arteriosus (PDA) and associated endocardial fibroelastosis. The descending aorta was fed exclusively by massive systemic collateral circulation.

A 45-year-old man came to our outpatient clinic because of blood pressure level abnormality (200/100 mm Hg), which had persisted for >30 years. The patient was found with abnormal blood pressure when he was 10 years old. Maximal blood pressure level at 260/110 mm Hg was recorded during the physical examination for several years. The patient had a smoking history of 20 years and no similar family medical history was reported, but suffers no other significant clinical symptoms. In recent weeks, he suffered dizziness and was treated with oral antihypertensive medications. The treatment regime was nifedipine (controlled release tablets 30 mg Bid), irbesartan and hydrochlorothiazide (tablets 150 mg qd), and irbesartan (150 mg qd). The curative effect is limited and his blood pressure remains at elevated level. On physical examination, the patient had a pulse rate of 68 bpm, a respiratory rate of 18 bmp, a body temperature of 36.7°C, and a body mass index of 23 kg/m^2^. The first medical evaluation in our hospital revealed a regular heart rate of 68 bpm and a grade 4/6 systolic murmur at the position of T1-T3 on back. The murmur conducted to neck. His blood pressure was measured as 183/92 mm Hg in the left arm and 175/95 mm Hg in the right arm, 96/64 mm Hg in the left ankle, and 101/60 mm Hg in the right ankle (left ankle brachial index: 0.54, right ankle brachial index: 0.56). It indicated a significant differential blood pressure between the upper and lower limbs. Echocardiography showed the left ventricle did not present severe abnormal dimensions and impaired systolic function, the descending aortic arch could not be observed, and aortic valve regurgitated moderately. Computed tomography angiogram (CTA) revealed a type A interrupted aortic arch with complete discontinuity of the aortic lumen distal to the origin of the left subclavian artery, and a massive aorta arch which included 2 full-fledged collaterals network ensuring blood circulation to distal aorta (Fig. 1). Angiography showed absence of anatomical continuity between the ascending aorta distal to the left subclavian artery and the descending aorta. There was no patent ductus arteriosus (PDA) and associated endocardial fibroelastosis. The descending aorta was fed exclusively by massive systemic collateral circulation (Fig. 2). Cardiac magnetic resonance (CMR) found nothing abnormal. Carotid artery ultrasonography indicated bilateral carotid plaques. Lower limbs (right) ultrasonography indicated plaques on the arterial wall. Based on the available evidences, we have concluded that the patient suffered from secondary hypertension due to isolated interrupted aortic arch (IAA). We had discussed the advantages and disadvantages of the surgery option with the patient, but he refused. Therefore, we had prescribed antihypertensive medications: nifedipine controlled release tablets (Adalat) 30 mg bid; irbesartan and hydrochlorothiazide tablets (Coaprovel) 150 mg qd; irbesartan (Aprovel) 150 mg qd; doxazosin mesylate extended release tablets (Cardura) 4 mg qn; spironolactone tablets (Antisterone) 20 mg qd; carvedilol tablets 5 mg bid.

**Figure 3:**
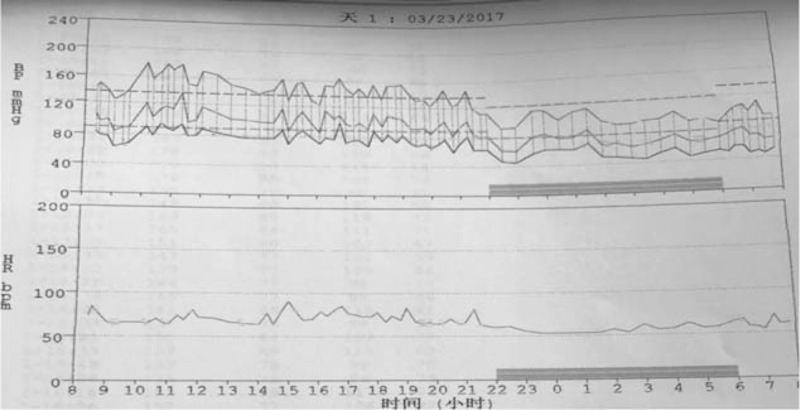
Twenty-four-hour ambulatory blood pressure monitoring (ABPM) revealed a mean of 24-h blood pressure at 129/68 mm Hg. The day and night mean blood pressure was 139/79 and 103/54 mm Hg, respectively.

After 10-day medication, 24-hour ambulatory blood pressure monitoring showed a mean of 24-hour blood pressure at 129/68 mm Hg. The day and night mean blood pressure was 139/79 and 103/54 mm Hg, respectively (Fig. 3). The mean office blood pressure fluctuated between 140 to 160 and 90 to 100 mm Hg. The significant discrepancy between ambulatory and office blood pressure should be attributed to the white coat effect.

## 4 Discussion

The physical examination is important in the diagnosis of secondary hypertension. In our case, the abnormal murmur indicates the possibility of congenital malformation which is a rare reason for refractory hypertension. Aortic arch interruption is classified into 3 type: type A is where interruption occurs distal to the left subclavian artery (as in our case); type B is where interruption occurs distal to the origin of left common carotid artery; and type C is where interruption occurs proximal to the origin of left common carotid artery. All reported adult cases are type A.^[6–10]^ It is very rare that the patient with IAA survived to 45 years of age. Formation of arterial collaterals leads our patient to survive into adulthood without other significant clinical complications. Interruption of the aortic arch may have allowed for collateral vessels formation during the fetal life, allowing sufficient blood supply to the lower part of the body. Commonly, the blood pressure of patients with IAA is uncontrollable with medications alone, but the ambulatory blood pressure monitoring (ABPM) for this patient showed normal level with the medication treatment. Restoration of arterial anatomy may be helpful to normalize blood pressure and ensure lower limbs blood supply. The patient's physical condition is sound, indicating that surgery outcome could be promising. Unfortunately, he refused the surgical treatment due to the concern of the additional risk associated with surgery. We will follow the patient and educate him on the accurate way of measuring blood pressure. If conditions are exacerbated, timely surgical therapy is highly recommended.^[2–5]^

## Acknowledgments

We gratefully acknowledge the technical assistance of Li-Jiang Tang and Ping Mao (Zhejiang Hospital, Zhejiang Province, China).
